# Neuroprotective Effects of ZiBuPiYin Recipe on db/db Mice via PI3K-Akt Signaling Pathway by Activating Grb2

**DOI:** 10.1155/2021/8825698

**Published:** 2021-01-30

**Authors:** Wei-ming Ren, Ze-bin Weng, Xin Li, Li-bin Zhan

**Affiliations:** ^1^School of Traditional Chinese Medicine & School of Integrated Chinese and Western Medicine, Nanjing University of Chinese Medicine, Nanjing 210023, China; ^2^Nanjing Hospital of Chinese Medicine Affiliated to Nanjing University of Chinese Medicine, Nanjing 210023, China; ^3^Dalian Medical University, Dalian, Liaoning 116044, China

## Abstract

**Background:**

Diabetes-associated cognitive decline (DACD) is one of the nervous system dysfunctions induced by diabetes mellitus with cognitive impairment as the major symptom. In a previous preliminary proteomic study, we found that endoplasmic reticulum processing and PI3K-Akt signaling pathway might be impaired in DACD pathogenesis. In addition, growth factor receptor-bound protein 2 might be a crucial protein as a molecular target of the neuroprotective effects of ZiBuPiYin recipe (ZBPYR).

**Methods:**

In this study, 6-8 weeks aged db/db mice were treated with excipients or ZBPYR for 6 weeks. Body weight and RBG were recorded weekly. Oral glucose tolerance and insulin tolerance tests were used to assess insulin sensitivity. Morris water maze (MWM) tests were used to assess memory function. The expression of Grb2, Gab2, Akt, and GSK3*β* in mouse hippocampus and cerebral cortex were analyzed by Western blotting.

**Results:**

ZBPYR not only significantly reduced RGB and improved glucose tolerance and insulin resistance, but also improved spatial cognition in DACD mice. The expression of Grb2 and Gab2 in hippocampus and cerebral cortex of db/db mice was upregulated after treated with ZBPYR, and then affected the PI3K/Akt signaling pathway, and inhibited GSK3*β* overactivity.

**Conclusions:**

This study showed that ZBPYR could enhance the memory and learning ability of db/db mice. Such neuroprotective effect might be related to the activation of Grb2-PI3K/Akt signaling which might provide a novel therapeutic target for the clinical treatment of DACD.

## 1. Introduction

Diabetes mellitus (DM) is the most common chronic metabolic disease with a high global prevalence. The global prevalence of diabetes has been increasing over recent decades. It was estimated that there were approximately 451 million people with DM worldwide in 2017 and was predicted to rise to 700 million in 2045 [1]. Diabetes-associated cognitive decline (DACD), also known as diabetes encephalopathy, is one of the most common neurological complications in DM patients [2]. The brain aging and neurodegenerative processes of DACD are similar to those of Alzheimer's disease (AD) [3]. Recent epidemiological studies suggested that people with DM have been associated with an increased risk of cognitive decline and dementia, including AD [4]. However, at present, the underlying pathophysiological mechanisms of DACD are not fully understood, and there is still no efficient cure or prevention [5]. Therefore, it is of great significance to study the pathogenesis of DACD and explore effective treatment options [6]. Animal studies may help to identify the mechanisms that underlie the adverse impact of diabetes on cognition and advance our understanding of its pathophysiology and may provide potential treatment on DACD. The leptin receptor-deficient db/db mice, one of the most widely used type 2 diabetes mellitus (T2DM) animal models, showed a series of diabetic symptoms such as obesity, hyperglycemia, hyperinsulinemia, insulin resistance, and diabetic complications like DACD as leptin plays a critical role in memory and learning [7, 8]. Therefore, db/db mice were used as DACD animal model in this study.

Traditional Chinese medicine (TCM) provides unique advantages in the treatment of complex metabolic diseases such as diabetes and its complications due to its multicomponent and multitarget characteristics [9]. ZiBuPiYin recipe (ZBPYR) is derived from a modification of ZiCheng decoction, a TCM recipe recorded in a TCM monograph named Bujuji written by Wu Cheng during Qing dynasty. Our preliminary study suggested that ZBPYR improved learning and memory ability in multiple animal models of diabetes [10–12]. We also found that ZBPYR protected hippocampal neurons against glutamate and amyloid beta-peptide- (A*β*-) induced neurotoxicity through blocking the serum-inducible kinase and spine-associated Rap GTPase-activating protein (SNK-SPAR) pathway [13, 14]. Moreover, in a previous preliminary proteomic study, we found that endoplasmic reticulum processing and PI3K-Akt signaling pathway might be impaired in DACD pathogenesis. In addition, growth factor receptor-bound protein 2 (Grb2) might be a crucial protein as a molecular target of the neuroprotective effects of ZBPYR [15].

Grb2 is an adaptor protein that transmits downward growth factor signals, consisting of an SH2 domain flanked by *N*- and *C*-terminal SH3 domain [16]. Grb2 recruits and mediates the interaction with the adaptor protein Grb2-associated binder Gab2, and the SH2 domain could bind tyrosine-phosphorylated proteins, such as tyrosine-phosphorylated Gab2 [17, 18]. Gab2 has been reported as an activator of phosphatidylinositol 3-kinase (PI3K) signaling pathway, since the interaction of SH2 domain of Grb2 with Gab2 recruited p85, which is the regulatory subunit of PI3K [19–21]. It has been demonstrated that PI3K activates its downstream effector Akt, which then promotes glycogen synthasekinase-3 (GSK3) phosphorylation [22]. Impaired PI3K/Akt/GSK3*β* signaling pathway modulates abnormal hyperphosphorylation of Tau protein which is one of the most important pathological lesions in AD [23–25]. The pathology of DACD is particularly similar to that of AD. Therefore, based on our previous study, we hypothesized that the cognitive dysfunction in diabetic mice might be related to the inhibition of the PI3K/Akt signaling pathway and neuroprotective effects of ZBPYR may be related to its upregulation of Grb2 in the brain and then activation of PI3K/Akt signaling pathway.

In this study, db/db mice (6 weeks old of age) were utilized to explore the improvement in memory and learning ability of ZBPYR and investigate its impact on Grb2 and PI3K/Akt signaling in the brain of db/db mice in order to interpret the neuroprotective effects and related mechanisms of ZBPYR.

## 2. Materials and Methods

### 2.1. Reagents

Primary antibodies p-Tyr452 Gab2 (#3882, 1 : 1000), PI3 kinase p85 (#4257, 1 : 1000), Akt (#9272, 1 : 1000), p-Ser473Akt (#4058, 1 : 1000), p-Ser9GSK3*β* (#9323, 1 : 1000), and beta-actin (#3700T, 1 : 2000) were purchased from Cell Signaling Technologies (Danvers, MA, USA). Primary antibody directed against Grb2 (#ab32111, 1 : 1000) and GSK3*β* (#ab131356, 1 : 1000) were from Abcam PLC (Cambridge, UK). Primary antibody Gab2 (sc-365590, 1 : 500) was from Santa Cruz (Santa Cruz, USA). Secondary antibodies were goat anti-rabbit (BA1054, 1 : 2000) (Boster Biological Technology Co. Itd, Wuhan, China) or anti-mouse (BA1050, 1 : 2000).

### 2.2. Animals

Male C57BLKS/J-db/db mice (6 weeks old of age) and age-matched nondiabetic littermates' db/m mice were obtained from Nanjing Qingzilan Technology Co., Ltd. (Nanjing, Jiangsu Province, China). Mice were housed in the Specific Pathogen Free (SPF) Animal Laboratory of Dalian Medical University. Specifically, mice were housed in a 12 h light/dark cycle (24°C ± 2°C and 65% ± 5% humidity) and received food and water ad libitum. All animal experiments were conducted in accordance with the NIH Principles of Laboratory Animal Care and the institutional guidelines for the care and use of laboratory animals at Dalian Medical University. All experiments were approved by the Committee on the Ethics of Animal Experiments of Dalian Medical University. All surgery was performed under anesthesia with ether (the usage of ether was approved by the Committee on the Ethics of Animal Experiments of Dalian Medical University), and all efforts were made to minimize suffering.

### 2.3. Preparation and Administration of ZBPYR

ZBPYR is consists of 12 Chinese Herbal Medicines: red ginseng (Radix Ginseng Rubra), common yam rhizome (Rhizoma Dioscoreae Oppositae), Indian Buead (PORIA), white peony root (Radix Paeoniae Alba), Dan shen root (Radix Salviae Miltiorrhizae), white hyacinth bean (Semen Lablab Album), lotus seed (Semen Nelumbinis), grassleaf sweetflag rhizome (Rhizoma Acori Tatarinowii), thinleaf milkwort root (Radix Palygalae), sandalwood (Lignum Santali Albi), tangerine red epicarp (Exocarpium Citri Rubrum), and liquorice root (Radix Glycyrrhizae). All herbs were purchased from Dalian Metro Pharmaceutical Co., Ltd., Dalian, Liaoning, China (the drug meets the 2015 National Pharmacopoeia Standard). The mixtures were soaked in distilled water (Milli-Q Integral Water Purification System, Millipore Corporation, Billerica, MA, USA) for 30 min and boiled in 8 volumes of water (v/w) for 90 min. The decoction was then concentrated to 3.29 g/ml and finally stored at 4°C.

We had done some works about the chemical components and quality control of the ZBPYR in the previous studies. For the quality control of ZBPYR, we combined high-performance liquid chromatography (HPLC) and electrospray ionisation quadrupole time-of-flight tandem mass spectrometry (HPLC-Q-TOF-MS) for fingerprint analysis and qualitative analysis. Seven common peaks (liquiritin, naringin, 3,6-disinapoylsucrose, 3,4,5-trimethoxycinnamic acid, rosmarinic acid, isoliquiritin apioside, and salvianolic acid B) were identified and detected [26]. In this experiment, ZBPYR was prepared according to the process described in the previous paper.

After one week of adaptive feeding, the db/db and db/m mice were randomly assigned to three groups: control group, DM group, and DM/ZBPYR group with 4 mice in each group. At 6th week, oral administration of ZBPYR treated with DM/ZBPYR group at a dose of 10 ml/kg body weight per day, while an identical dose of ultrapure water (Milli-Q Integral Water Purification System, Millipore Corporation, Billerica, MA, USA) was administered to DM and control groups.

### 2.4. Random Blood Glucose and Fasting Serum Insulin

Body weight and random blood glucose (RBG) were measured weekly during the whole treatment to verify the development of diabetes in the db/db mice. At the end of the administration, mice were starved for 12 h and blood samples were obtained from the inner canthus vein of the eye after being anesthetized with ether. Blood samples were then centrifuged (4°C, 3000 rpm) for 15 min, and serum samples were obtained and stored at -80°C until measuring fasting serum insulin (FSI). FSI levels were assayed using an insulin radioimmunoassay kit (Atom High-tech, Beijing, China).

### 2.5. Oral Glucose Tolerance Test and Insulin Tolerance Test

Oral glucose tolerance test (OGTT) and insulin tolerance test (ITT) were performed at the end of the administration period. Mice received oral administration of 2 g/kg body weight of 50% (wt/wt) glucose solution after fasted for 14 h. Blood samples were collected at 0, 30, 60, and 120 min from tail vein after glucose administration. In insulin tolerance test, mice were injected intraperitoneally with regular human insulin (Novolin, Novo Nordisk Pharmaceutical Co., Ltd., Tianjin, China) at a dose of 0.75 U/kg body weight after fasted for 6 h. Glucose levels were monitored at 0, 15, 30, 60, 90, and 120 min after insulin injection. OGTT and ITT were determined using a strip-operated blood glucose sensor (Roche, Mannheim, Germany).

### 2.6. Morris Water Maze Test

Morris water maze test was performed for 6 days to assess spatial learning and memory performance of the mice. The water maze is consisted of a round tank (Institute of Materia Medica, Chinese Academy of Medical Sciences, Beijing, China), which was 100 cm in diameter, 50 cm in height, and filled with water and milk powder maintained at 26°C ± 1°C, a transparent platform, which was 9 cm in diameter, 29 cm in height, and located at 30 cm from the edge of the tank and hidden under water, and an automatic photographic recording and analysis system (EthoVision, Noldus Information Technology b.v., Wageningen, Netherlands). On the 1st day, mice were permitted to swim freely in the pool for 120 s without the platform. During the following 4 days, mice were trained on 4 trials per day at intervals of 60 s. The platform location was submerged 1 cm below the water surface, and the starting points were changed every trial. Each trial lasted until the mice swam to the platform or for a maximum observation time of 120 s. If the mice failed to swim to the platform within 120 s, they were guided to the platform. On the day after the last acquisition training session, mice were tested in a single 120 s probe test without the platform. Over this period, time in seeking the platform location (escape latency), time in the target quadrant where the platform had been located, and the number of platform location crossings were all measured automatically. On the 6th day, mice were trained on a visible-platform test. The platform was located 1 cm over the water surface and fixed at a position different from the previous ones. During this test, the experimental procedures were performed as same as previous tests, and escape latency and swimming distance were recorded.

### 2.7. Sample Preparation

The mice were anesthetized with ether and decapitated for Western blotting analysis. Hippocampus and cerebral cortex were rapidly dissected via surgery on ice. All samples were immediately frozen in liquid nitrogen and stored at -80°C until required. Hippocampus and cerebral cortex samples were homogenized in ice-cold lysis buffer (0.125 M Tris HCl (pH 6.8), 0.2 M DTT, 4% SDS, and 20% glycerol). The lysates were sonicated for 10 min and centrifuged (4°C and 15,000 rpm) for 5 min to remove insoluble debris. Protein concentrations in the supernatants were measured by a Minim Spectrophotometer (NanoVueTM Plus, GE Healthcare, Amersham Place, Little Chalfont, Buckinghamshire, HP79NA, UK).

### 2.8. Western Blotting

Protein (50 mg per sample) was loaded per line and separated on 8%–15% tris-glycine polyacrylamide gels and transferred to nitrocellulose membranes. Immunoblots were blocked for 2 h in Tris-buffered saline Tween-20 (TBST, 20 mM Tris-HCl, 150 mM NaCl, pH 7.5, and 0.05% Tween 20) containing 5% skim milk. The blots were then incubated with primary antibodies in TBST at 4°C overnight. Membranes were washed three times with TBST and then incubated with secondary antibody for 2 h at room temperature. Membranes were washed again and developed with enhanced chemiluminescence (ECL) and detected with X-ray films. The blots were visualized with ImageQuant TL 1D (GE Healthcare, USA). Primary antibodies used from Cell Signaling Technologies (Danvers, MA, USA) were as follows: p-Tyr452 Gab2 (#3882, 1 : 1000), PI3 kinase p85 (#4257, 1 : 1000), Akt (#9272, 1 : 1000), p-Ser473Akt (#4058, 1 : 1000), p-Ser9GSK3*β* (#9323, 1 : 1000), and beta-actin (#3700T, 1 : 2000). Primary antibody directed against Grb2 (Abcam PLC, Cambridge, UK, #ab32111, 1 : 1000), GSK3*β* (Abcam PLC, Cambridge, UK, #ab131356, 1 : 1000), and Gab2 (Santa Cruz, USA, sc-365590, 1 : 500) were also used in the experiments. Secondary antibodies were goat anti-rabbit (BA1054, 1 : 2000) (Boster Biological Technology Co. Itd, Wuhan, China) or anti-mouse (BA1050, 1 : 2000).

### 2.9. Statistical Analysis

Statistical analysis was performed using SPSS 17.0. All experimental data were statistically analyzed by one-way ANOVA and Student's *t*-test to compare all groups. The difference was considered to be statistically significant when *p* < 0.05.

## 3. Results

### 3.1. Effects of ZBPYR on Peripheral Glucose Homeostasis and Insulin Sensitivity

**Figure 1:**
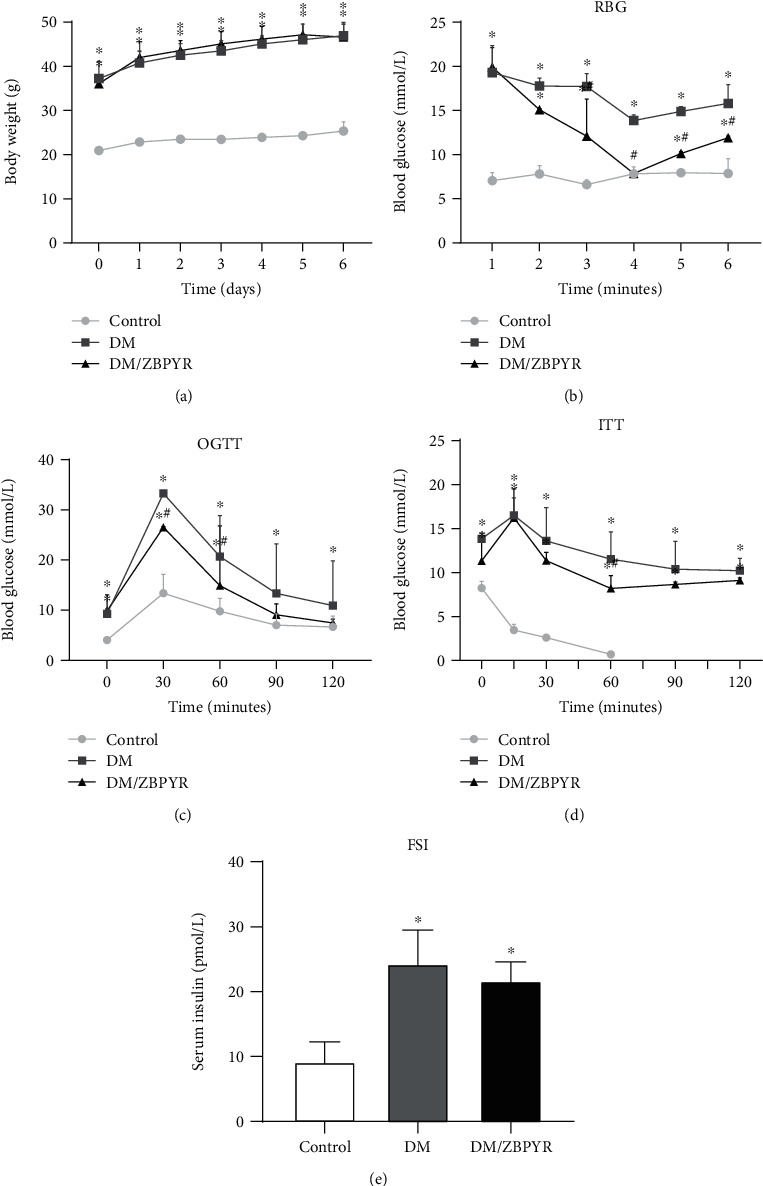
The antidiabetic effects of ZBPYR on db/db mice. (a) Body weight was recorded weekly. (b) RBG level was measured every week in the three groups during ZBPYR administration. (c–e) OGTT, ITT and FSI level were measured at the end of treating period. Values are means ± S.D. from 4 mice in each group. ^∗^*p* < 0.05 compared to control; ^#^*p* < 0.05 compared to DM.

As shown in Figure 1(a), body weight of DM mice was significantly heavier compared to control mice while ZBPYR administration had no significant effects on body weight. RBG levels of DM/ZBPYR mice were significantly lower than DM mice from the 3rd week to the 6th week of treatment (Figure 1(b)). The OGTT results revealed that ZBPYR significantly reduced the blood glucose levels at 30 min (0.80 times) and 60 min (0.72 times) after glucose load (Figure 1(c)). Additionally, the administration of ZBPYR also significantly decreased the blood glucose levels at 60 min (0.64 times) after insulin injection (Figure 1(d)). DM mice exhibited impaired glucose tolerance and insulin sensitivity which are associated with the development of cognitive decline and poor cognitive performance [27]. Treatment with ZBPYR could improve the impaired glucose tolerance and insulin sensitivity of DM mice. However, there was no significant difference in FSI levels between DM/ZBPYR and DM mice (Figure 1(e)).

### 3.2. ZBPYR Improves Spatial Learning and Memory Performance

**Figure 2:**
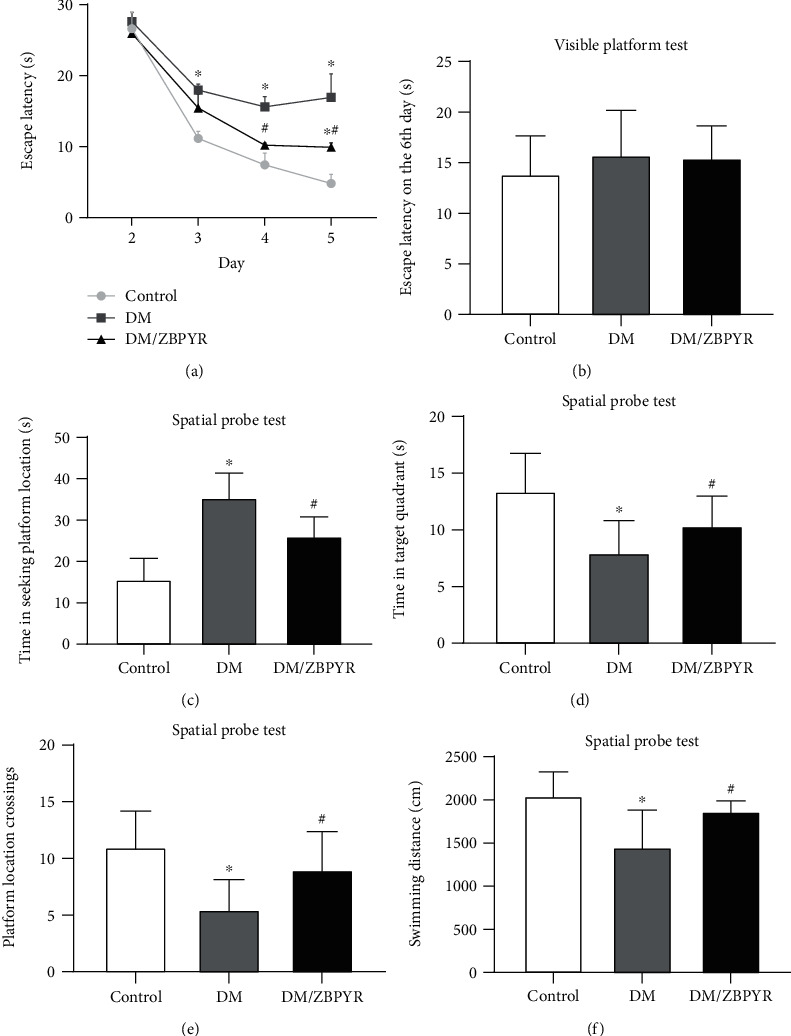
Effects of ZBPYR on DACD mice in Morris water maze test. (a) escape latency, (b) escape latency in the visible platform test, (c) time in searching original platforms, (d) time in target quadrant, (e and f) platform location crossings and swimming distance in spatial probe test. Values are means ± S.D. from 4 mice in each group. ^∗^*p* < 0.05 compared to control; ^#^*p* < 0.05 compared to DM.

Lesions in animals' brain regions, such as hippocampus and cerebral cortex, have an impairment on Morris water maze performance [22]. The results of Morris water maze test revealed that DM mice required significantly longer escape latency than the control ones between day 3 and day 5 (day 3 1.92 times, day 4 2.31 times, and day 5 5.41 times, respectively). The latency of DM/ZBPRY mice was shorter than that of DM mice on day 4 (0.70 times) and day 5 (0.59 times) (Figure 2(a)). In the visible platform test on the 6th day, there was no significant difference among the three groups in escape latency (Figure 2(b)).

In the spatial probe test, the swimming time of DM/ZBPYR mice in seeking platform location was shorter (0.75 times) than that of DM mice (Figure 2(c)). And the moving time of DM/ZBPYR mice in the target quadrant was longer (1.25 times) than that of DM mice (Figure 2(d)). DM/ZBPYR mice exhibited more times (1.72 times) of crossing the platform location than DM mice (Figure 2(e)). Also, the swimming distance of DM/ZBPYR mice was enhanced 1.29 times compared to DM mice (Figure 2(f)).

### 3.3. ZBPYR Administration Increases the Expressions of Grb2 and Gab2 and Affects PI3K/Akt Signaling Pathway in the Hippocampus of db/db Mice

**Figure 3:**
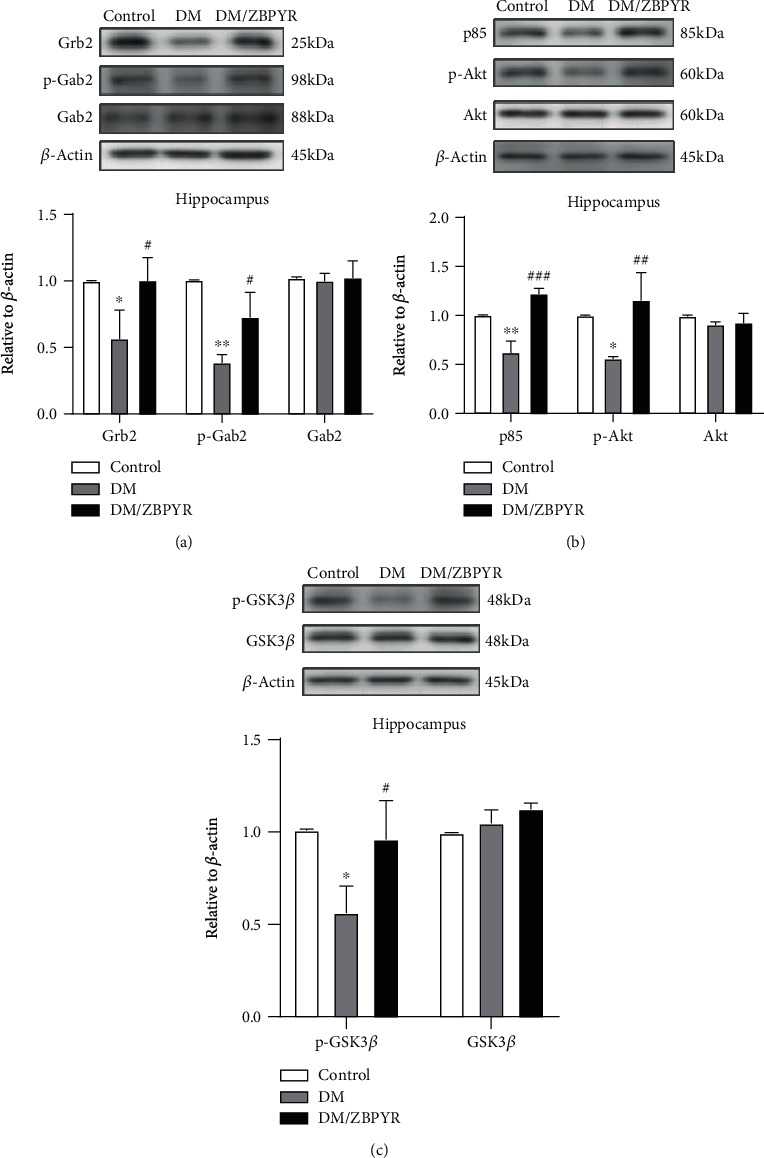
The effect of ZBPYR on the expression of Grb2, Gab2, Akt, p-Akt, and GSK3*β* in mouse hippocampus. (a) Compared with the control group, the expression of Grb2 in the hippocampus of the DM group was reduced. However, Grb2 levels increased after ZBPYR administration. The total level of Gab2 in the hippocampus did not change significantly between the three groups. The expression of p-Gab2 (Tyr452) in the hippocampus of DM/ZBPYR mice was moderately increased. (b) The levels of p85 and p-Akt (Ser473) were reduced in DM, while ZBPYR enhanced p85 and Akt hyperphosphorylation. The total Akt level was not different in the hippocampus between the three groups. (c) DM group hippocampus Ser9-phosphorylated GSK3*β* decreased, while p-GSK3*β* (Ser9) levels in the DM/ZBPYR group hippocampus increased. Values are means ± S.D. from 3 mice in each group. ^∗^*p* < 0.05 and ^∗∗^*p* < 0.01 compared to control; ^#^*p* < 0.05, ^##^*p* < 0.01, and ^###^*p* < 0.01 compared to DM.

The hippocampus plays an especially important role on cognition and memory in mammals [28]. The result showed that expressions of Grb2, Gab2, p85, and Akt were different in hippocampus among the three groups. An obvious reduction in Grb2 expression was observed in the hippocampus of DM group compared with control group. Treatment with ZBPYR could significantly increase Grb2 expression. In addition, the expression of Gab2 showed no significant changes among the three groups (Figure 3(a)). As Tyr452 of Gab2 is a potential binding site of p85, the regulatory subunit of PI3 kinase [29], we detected the expression of p-Gab2 (Tyr452). Results revealed that the expression of p-Gab2 (Tyr452) modestly increased in the hippocampus of DM/ZBPYR mice compared to DM mice (Figure 3(a)). Moreover, ZBPYR affected PI3K/Akt signaling pathway in the hippocampus of db/db mice. Specifically, the levels of p85 and p-Akt (Ser473) were both decreased in the hippocampus of DM (Figure 3(b)) when compared to the control group, while ZBPYR treatment could enhance p85 (Figure 3(b)) and Akt hyperphosphorylation (Figure 3(b)).

### 3.4. ZBPYR Increases the Expressions of Grb2 and Gab2 and Affects PI3K/Akt Signaling Pathway in the Cerebral Cortex of db/db Mice

**Figure 4:**
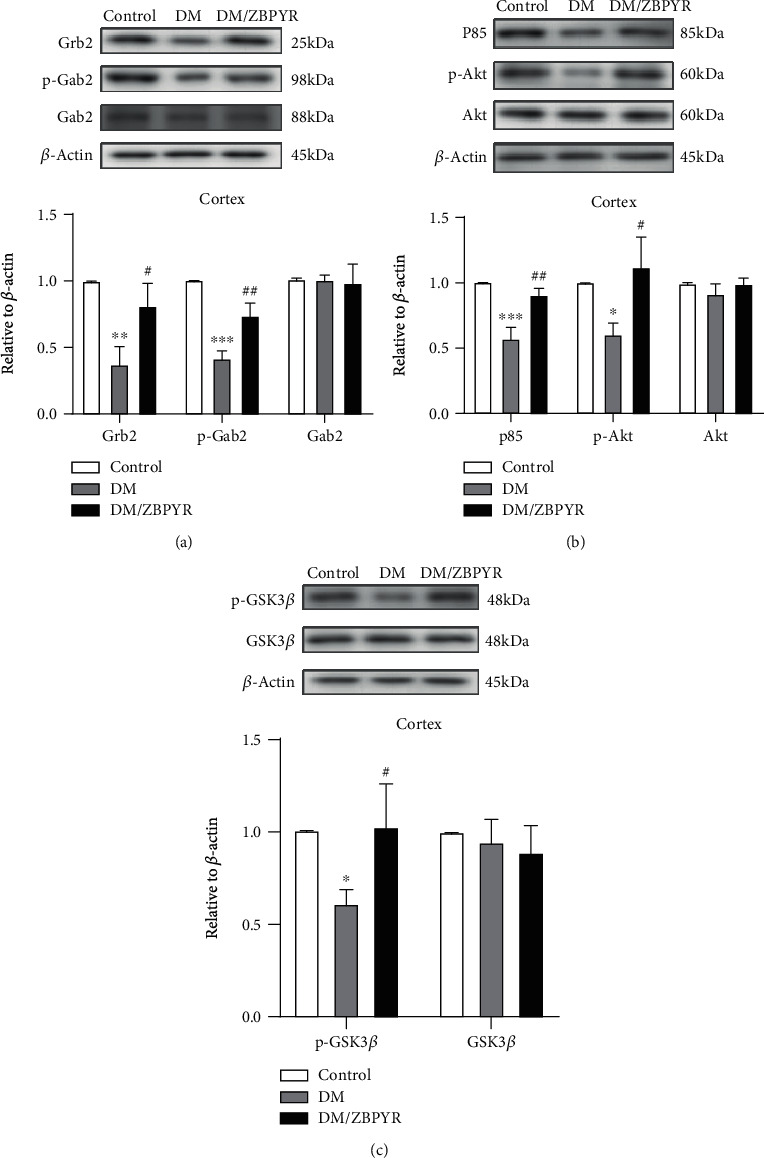
The effect of ZBPYR on the expression of Grb2, Gab2, Akt, p-Akt, and Gsk3*β* in mouse cerebral cortex. (a) The expression of Grb2 and p-Gab2 (Tyr452) in the cerebral cortex of DM mice was significantly reduced, and Grb2 and p-Gab2 (Tyr452) were increased to varying degrees after ZBPYR treatment. There were no statistically significant changes in the total levels of Gab2 and Akt in the three groups. (b) Compared with control mice, p-Akt (Ser473) expression in the cerebral cortex of DM mice was significantly reduced, while that of DM/ZBPYR mice was significantly increased. Compared with the DM group, p85 was significantly increased in the cerebral cortex of the DM/ZBPYR group. (c) The degree of Ser9 phosphorylation of GSK3*β* in the cerebral cortex of the DM group was reduced to varying degrees, and the level of p-GSK3*β* (Ser9) was increased in the cerebral cortex of the DM/ZBPYR group. Values are means ± S.D. from 3 mice in each group. ^∗^*p* < 0.05, ^∗∗^*p* < 0.01, and ^∗∗∗^*p* < 0.01 compared to control; ^#^*p* < 0.05 and ^##^*p* < 0.01 compared to DM.

The cerebral cortex is also closely related to cognitive function [30, 31]. In this study, we did observe an obvious reduction in Grb2 and p-Gab2 (Tyr452) expressions in the cerebral cortex of DM mice compared to control (Figure 4(a)), and ZBPYR showed an ability to provide Grb2 and p-Gab2 (Tyr452) with varying degrees of increase (Figure 4(a)). Similarly, results revealed no statistically significant changes in the total levels of Gab2 (Figure 4(a)) and Akt (Figure 4(b)) in the cerebral cortex among the three groups. However, an apparent reduction in expression of p-Akt (Ser473) was detected in the cerebral cortex of DM mice when compared with control mice (Figure 4(b)), with an evident increase in DM/ZBPYR mice (Figure 4(b)). We also observed a significantly increased p85 in the cerebral cortex of DM/ZBPYR group compared to DM group (Figure 4(b)).

### 3.5. ZBPYR Inhibits GSK3*β* Overactivity

It has been clear that PI3K/Akt signaling pathway affects GSK3*β* activity in the brain [32]. In our study, we observed the alteration of GSK3*β* activity. However, we did not detect the statistically significant alterations in total GSK3*β* expressions among the three groups. Additionally, Western blotting results showed different extent of reduction in phosphorylated GSK3*β* at Ser9 in the hippocampus (Figure 3(c)) and cerebral cortex (Figure 4(c)) of DM group, while the levels of p-GSK3*β* (Ser9) increased in the hippocampus (Figure 3(c)) and cerebral cortex (Figure 4(c)) of DM/ZBPYR group.

## 4. Discussion

As DACD is a common complication of diabetes, the pathogenesis and effective treatment strategies of DACD have attracted increasing attention from researchers. DACD is associated with blood glucose and serum insulin level. Pharmaceutical therapy targeted on diabetic symptoms such as hyperglycemia, hyperinsulinemia, and insulin resistance and also treats some diabetic complications [33, 34]. Many studies have revealed that TCM has antidiabetic effects, providing multiple therapeutic effects on blood glucose and serum insulin level [35]. Here, we explored the effects of ZBPYR on blood glucose and serum insulin level. The results revealed that ZBPYR exhibited promising antidiabetic effects by moderating the increased RBG levels during the 3rd to 6th weeks of treatment and significantly improving the impaired glucose tolerance and insulin sensitivity.

Morris water maze test is widely used for testing spatial learning and memory [36]. T2DM mice exhibited severe cognitive deficits, seriously impair their Morris water maze performance [37]. Our results demonstrated that DM mice had severe cognitive impairment, and ZBPYR was able to improve these cognitive impairment and inferior learning and memory performance of DM mice, which could be seen in the training test (4th and 5th days) and spatial probe test.

Our previous studies have found that endoplasmic reticulum processing and PI3K-Akt signaling pathway might be impaired in DACD pathogenesis [15]. In addition, Grb2 might be a crucial protein as a molecular target of the neuroprotective effects of ZBPYR. The PI3K/Akt signaling pathway regulates cell proliferation, differentiation, metabolism, and cytoskeletal reorganization, leading to apoptosis and cancer cell survival. The PI3K/Akt pathway is the main pathway for insulin signal transduction and involved in the regulation of blood glucose [38]. Grb2 is a cytoplasmic connexin, and studies have shown that Grb2 can prevent AD by binding to insulin receptors to improve the expression of downstream molecules of insulin signaling to prevent diabetic peripheral neuropathy and interacting with NOX4 to protect the cytoskeletal disassembly [39, 40].

These studies indicated that Grb2 might play a key role in degenerative neuropathy. In the present study, we investigated the expression of Grb2, Gab2, p85, Akt, and GSK3*β* in the hippocampus and cerebral cortex, which are associated with cognitive function in humans and animals. Western blotting analysis results showed that ZBPYR affects the PI3K/Akt signaling pathway and increases the expression of p85 and p-Akt (Ser473) by enhancing the expression of Grb2 and Gab2 hyperphosphorylation in the hippocampus and cerebral cortex of db/db mice. Our study also found that ZBPYR could inhibit the downstream GSK3*β* over activity. All these results indicated that ZBPYR exerts a neuroprotective effect via PI3K-Akt signaling pathway by activating Grb2 on the brain of db/db mice.

## 5. Conclusions

In the present study, we investigated the neuroprotective effects of ZBPYR on db/db mice. The results demonstrated that ZBPYR could improve the learning and cognitive functions in DACD mice. The underlying mechanism may be related to the regulation of PI3K-Akt signaling pathway by activating Grb2.

## Data Availability

The data used to support the findings of this study were supplied by Prof. Libin Zhan under license and so cannot be made freely available. Requests for access to these data should be made to Libin Zhan, zlbnj@njucm.edu.cn, School of Traditional Chinese Medicine & School of Integrated Chinese and Western Medicine, Nanjing University of Chinese Medicine, Nanjing 210023, China.

## Supplementary Materials

Supplementary Materials Figure S1. Uncropped image of the original western blotting for p-Gab2/Gab2/Grb2/P85/p-Akt/Akt/p-GSK3*β*/GSK3*β* of hippocampus. Figure S2. Uncropped image of the original western blotting for p-Gab2/Gab2/Grb2/P85/p-Akt/Akt/p-GSK3*β*/ GSK3*β* of cortex.

## References

[1] Cho N. H., Shaw J. E., Karuranga S. (2018). IDF Diabetes Atlas: global estimates of diabetes prevalence for 2017 and projections for 2045. *Diabetes Research and Clinical Practice*.

[2] Wu Y., Ye L., Yuan Y. (2019). Autophagy activation is associated with neuroprotection in diabetes-associated cognitive decline. *Aging and Disease*.

[3] Weuve J., Barnes L. L., Mendes de Leon C. F. (2018). Cognitive aging in black and white Americans: cognition, cognitive decline, and incidence of Alzheimer disease dementia. *Epidemiology*.

[4] Huang C. C., Chung C. M., Leu H. B. (2014). Diabetes mellitus and the risk of Alzheimer’s disease: a nationwide population-based study. *PLoS One*.

[5] Biessels G. J., Despa F. (2018). Cognitive decline and dementia in diabetes mellitus: mechanisms and clinical implications. *Nature Reviews. Endocrinology*.

[6] Tumminia A., Vinciguerra F., Parisi M., Frittitta L. (2018). Type 2 diabetes mellitus and Alzheimer’s disease: role of insulin signalling and therapeutic implications. *International Journal of Molecular Sciences*.

[7] Yermakov L. M., Griggs R. B., Drouet D. E. (2019). Impairment of cognitive flexibility in type 2 diabetic db/db mice. *Behavioural Brain Research*.

[8] Zheng Y., Yang Y., Dong B. (2016). Metabonomic profiles delineate potential role of glutamate-glutamine cycle in db/db mice with diabetes-associated cognitive decline. *Molecular Brain*.

[9] Zheng W., Wang G., Zhang Z., Wang Z., Ma K. (2020). Research progress on classical traditional Chinese medicine formula Liuwei Dihuang pills in the treatment of type 2 diabetes. *Biomedicine & Pharmacotherapy*.

[10] Bi T., Zhan L., Zhou W., Sui H. (2020). Effect of the ZiBuPiYin recipe on diabetes-associated cognitive decline in Zucker diabetic fatty rats after chronic psychological stress. *Frontiers in Psychiatry*.

[11] Chen J., Liang L., Zhan L. (2014). ZiBuPiYin recipe protects db/db mice from diabetes-associated cognitive decline through improving multiple pathological changes. *PLoS One*.

[12] Chen J., Zhan L., Lu X., Xiao C., Sun N. (2017). The alteration of ZiBuPiYin recipe on proteomic profiling of forebrain postsynaptic density of db/db mice with diabetes-associated cognitive decline. *Journal of Alzheimer's Disease*.

[13] Zhan L. B., Niu X. P., Sui H., Gong X. Y. (2009). Protective effect of spleen-yin-nourishing recipe on amyloid beta-peptide-induced damage of primarily cultured rat hippocampal neurons and its mechanism. *Zhong Xi Yi Jie He Xue Bao*.

[14] Zhan L. B., Sui H., Lu X. G., Sun C. K., Zhang J., Ma H. (2008). Effects of Zibu Piyin recipe (滋补脾阴方药) on SNK-SPAR pathway in neuron injury induced by glutamate. *Chinese Journal of Integrative Medicine*.

[15] Shi X., Lu X. G., Zhan L. B. (2011). The effects of the Chinese medicine ZiBu PiYin recipe on the hippocampus in a rat model of diabetes-associated cognitive decline: a proteomic analysis. *Diabetologia*.

[16] Liao T. J., Jang H., Nussinov R., Fushman D. (2020). High-affinity interactions of the nSH3/cSH3 domains of Grb 2 with the C-terminal proline-rich domain of SOS1. *Journal of the American Chemical Society*.

[17] Gu D. H., Mao J. H., Pan X. D. (2017). MicroRNA-302c-3p inhibits renal cell carcinoma cell proliferation by targeting Grb2-associated binding 2 (Gab2). *Oncotarget*.

[18] Teal H. E., Ni S., Xu J. (2006). GRB2-mediated recruitment of GAB2, but not GAB1, to SF-STK supports the expansion of Friend virus-infected erythroid progenitor cells. *Oncogene*.

[19] Tian J., Zhang H., Mu L. (2020). The miR-218/GAB2 axis regulates proliferation, invasion and EMT via the PI3K/AKT/GSK-3*β* pathway in prostate cancer. *Experimental Cell Research*.

[20] Wang C., Gu C., Jeong K. J. (2017). YAP/TAZ-mediated upregulation of GAB2 leads to increased sensitivity to growth factor-induced activation of the PI3K pathway. *Cancer Research*.

[21] Wang Y., Sheng Q., Spillman M. A., Behbakht K., Gu H. (2012). Gab2 regulates the migratory behaviors and E-cadherin expression via activation of the PI3K pathway in ovarian cancer cells. *Oncogene*.

[22] Hermida M. A., Dinesh Kumar J., Leslie N. R. (2017). GSK3 and its interactions with the PI3K/AKT/mTOR signalling network. *Adv Biol Regul*.

[23] Wang Y., Chang Q. (2020). MicroRNA miR-212 regulates PDCD4 to attenuate A*β*25–35-induced neurotoxicity via PI3K/AKT signaling pathway in Alzheimer’s disease. *Biotechnology Letters*.

[24] Xiong R., Wang X. L., Wu J. M. (2020). Polyphenols isolated from lychee seed inhibit Alzheimer’s disease-associated Tau through improving insulin resistance via the IRS-1/PI3K/Akt/GSK-3*β* pathway. *Journal of Ethnopharmacology*.

[25] Yao Y., Wang Y., Kong L., Chen Y., Yang J. (2019). Osthole decreases tau protein phosphorylation via PI3K/AKT/GSK-3*β* signaling pathway in Alzheimer’s disease. *Life Sciences*.

[26] Dong P., Zhang L., Zhan L., Liu Y. (2016). Ultra high performance liquid chromatography with mass spectrometry for the rapid analysis and global characterization of multiple constituents from Zibu Piyin recipe. *Journal of Separation Science*.

[27] Sun L., Diao X., Gang X. (2020). Risk factors for cognitive impairment in patients with type 2 diabetes. *Journal Diabetes Research*.

[28] Novellino F., Lopez M. E., Vaccaro M. G., Miguel Y., Delgado M. L., Maestu F. (2019). Association between Hippocampus, thalamus, and caudate in mild cognitive impairment APOE*ε*4 carriers: a structural covariance MRI study. *Frontiers in Neurology*.

[29] Strunk U., Ramos D. G., Saffran H. A., Smiley J. R. (2016). Role of Herpes simplex virus 1 VP11/12 tyrosine-based binding motifs for Src family kinases, p 85, Grb2 and Shc in activation of the phosphoinositide 3-kinase-Akt pathway. *Virology*.

[30] Mitchell A. S., Czajkowski R., Zhang N., Jeffery K., Nelson A. J. D. (2018). Retrosplenial cortex and its role in spatial cognition. *Brain and Neuroscience Advances*.

[31] Sambuchi N., Geda Y. E., Michel B. F. (2019). Cingulate cortex in pre-MCI cognition. *Handbook of Clinical Neurology*.

[32] Ozaita A., Puighermanal E., Maldonado R. (2007). Regulation of PI3K/Akt/GSK-3 pathway by cannabinoids in the brain. *Journal of Neurochemistry*.

[33] Bilal M., Iqbal M. S., Shah S. B., Rasheed T., Iqbal H. M. N. (2018). Diabetic complications and insight into antidiabetic potentialities of ethno-medicinal plants: a review. *Recent Patents on Inflammation & Allergy Drug Discovery*.

[34] Yaribeygi H., Butler A. E., Barreto G. E., Sahebkar A. (2018). Antioxidative potential of antidiabetic agents: a possible protective mechanism against vascular complications in diabetic patients. *Journal of Cellular Physiology*.

[35] Zhang T. T., Jiang J. G. (2012). Active ingredients of traditional Chinese medicine in the treatment of diabetes and diabetic complications. *Expert Opinion on Investigational Drugs*.

[36] Bromley-Brits K., Deng Y., Song W. (2011). Morris water maze test for learning and memory deficits in Alzheimer’s disease model mice. *Journal of visualized experiments: JoVE*.

[37] Tang S. S., Ren Y., Ren X. Q. (2019). ER*α* and/or ER*β* activation ameliorates cognitive impairment, neurogenesis and apoptosis in type 2 diabetes mellitus mice. *Experimental Neurology*.

[38] Gao Y. F., Zhang M. N., Wang T. X., Wu T. C., Ai R. D., Zhang Z. S. (2016). Hypoglycemic effect of D-chiro-inositol in type 2 diabetes mellitus rats through the PI3K/Akt signaling pathway. *Molecular and Cellular Endocrinology*.

[39] Majumder P., Roy K., Singh B. K., Jana N. R., Mukhopadhyay D. (2017). Cellular levels of Grb2 and cytoskeleton stability are correlated in a neurodegenerative scenario. *Disease Models & Mechanisms*.

[40] Manu M. S., Rachana K. S., Advirao G. M. (2017). Altered expression of IRS2 and GRB2 in demyelination of peripheral neurons: implications in diabetic neuropathy. *Neuropeptides*.

