# Supplementary material for: Neuroprotective Effects of ZiBuPiYin Recipe on db/db Mice via PI3K-Akt Signaling Pathway by Activating Grb2
Source: Neural Plast. 2021 Jan 30;2021:8825698. doi: 10.1155/2021/8825698 (PMC7868140; doi:10.1155/2021/8825698)
Supplement: Supplementary Materials — Figure S1. Uncropped image of the original western blotting for p-Gab2/Gab2/Grb2/P85/p-Akt/Akt/p-GSK3β/GSK3β of hippocampus. Figure S2. Uncropped image of the original western blotting for p-Gab2/Gab2/Grb2/P85/p-Akt/Akt/p-GSK3β/ GSK3β of cortex. [file 8825698.f1.docx]

**Supplementary Materials**

**Figure S1.** Uncropped image of the original western blotting for p-Gab2/Gab2/Grb2/P85/p-Akt/Akt/p-GSK3β/GSK3β of hippocampus.

**Figure S2.** Uncropped image of the original western blotting for p-Gab2/Gab2/Grb2/P85/p-Akt/Akt/p-GSK3β/ GSK3β of cortex.
